# Asynchronous Bilateral Renal Infarction and Thrombophilia With Associated Gene Mutations in a 43-Year-Old Man: A Case Report

**DOI:** 10.1097/MD.0000000000003258

**Published:** 2016-04-08

**Authors:** Xu-Jie Zhou, Li-Jun Liu, Min Chen, Fu-De Zhou

**Affiliations:** From the Renal Division, Peking University First Hospital, Peking University Institute of Nephrology, Key Laboratory of Renal Disease, Ministry of Health of China, and Key Laboratory of Chronic Kidney Disease Prevention and Treatment (Peking University), Ministry of Education; Beijing, China.

## Abstract

Renal infarction (RI) is frequently misdiagnosed or diagnosed late because of its rarity and nonspecific clinical presentation, which may result in irreversible damage to the renal parenchyma or increase the risk of other embolic events affecting additional organs. Multiple causal mechanisms and cases of idiopathic RI have been reported, but the causal factors are not clear in most cases.

Here, we report the case of a patient with heterochronic bilateral RI caused by thrombophilia. Although he had several risk factors for hypercoagulation disorders, two gene mutations—*MTHFR* 677 C>T and *PLG* 1858G>A—were identified by genome sequencing of the entire exome. The findings suggest the possibility of a synergistic relationship between the two gene mutations.

Thus, screening for gene mutations may provide additional clues for clarifying the cause of RI and thrombophilia.

## Abstract 2

Supplemental Digital Content is available in the text

## INTRODUCTION

Renal infarction (RI) is an arterial vascular leading to irreversible renal damage. It is uncommon, with an estimated prevalence of 1.4% from autopsy studies and 0.004%–0.007% from emergency department visits.^4–6^ Because of its rarity and nonspecific clinical presentation, it is easily missed or misdiagnosed in most cases, as a result of which there is irreparable damage to the renal parenchyma and a greater risk of other embolic events affecting additional organs. The underlying mechanism is complex. To risk factors belong atrial fibrillation, valvular or ischemic heart disease, endocarditis, hypercoagulation, hematologic diseases, spontaneous renal artery dissection, etc.^1–4^ However, the causal factor is not clear in a considerable number of cases, so there is a need for exhaustive investigation on the cause of RI. Four types of RI based on etiologic factors were suggested: RI of cardiac origin, RI associated with renal artery injury, RI associated with hypercoagulability, and apparently idiopathic RI.^2,4^ Although inherited hypercoagulable states have also been implicated, to date, gene mutation analyses have been mainly candidate gene based. There are less than 10 reported cases with mutational analysis and all were from Caucasians (Supplementary Table 1).^2–6^ Reported mutations included MTHFR C677T mutation, antithrombin III deficiency, prothrombin gene mutation, and factor V Leiden mutation. Thus whole-genome-wide mutation screening is appropriate and mutational analysis in Asians will be of particular interest.

Here, we report a case of heterochronic bilateral RI caused by a prothrombotic disorder, in which the entire exome of the patient was sequenced. Two gene mutations—*MTHFR* (5,10-methylenetetrahydrofolate reductase gene) 677 C>T and *PLG* (plasminogen) 1858G>A—were identified as possible genetic determinants of the homocysteine level and dysplasminogenemia, respectively. The data suggests that gene mutation screening may provide additional clues for clarifying the cause of RI and thrombophilia.

## CASE REPORT

The patient was a 43-year-old male Chinese farm worker with heterochronic bilateral RI caused by a prothrombotic disorder. He had a 20-year history of smoking 20 pieces cigarette per day. He had no history of diabetes, dyslipidemia, or atrial fibrillation. Further, no similar disease history was observed in his family (no similar symptom of abdominal pain or abnormal findings in urine tests or in physical examinations).

Two years before presentation to our hospital, he was admitted to a local hospital with right-sided cutting pain in the abdomen. Apart from mild nausea and vomiting, there were no other relevant symptoms. He was diagnosed with acute appendicitis, for which he underwent appendectomy. After the operation, the pain persisted, and the patient additionally presented with incomplete ileus and fever. Urinalysis revealed that the urine protein score was 2+ and hematuria was absent. The serum creatinine level was 113.8 μmol/L (normal range, 44–110 μmol/L), and blood urea nitrogen (BUN) was 5.48 mmol/L. No significant abnormality in the kidneys was observed on a plain CT scan. He was orally administered an antacid, an analgesic, and antibiotics, but the abdominal pain persisted. He underwent Chinese acupuncture therapy for 2 weeks and the abdominal pain slowly subsided. Thereafter, he did not undergo any further tests for renal function or imaging examinations.

Nineteen days before presentation to our hospital, the patient had presented to another local hospital with acute pain in the left loin, nausea, and vomiting. He had a body temperature of 37.5°C and systolic blood pressure of 140 mm Hg. Blood analyses revealed left-shifted leukocytosis, mild elevation in alanine and aspartate aminotransferase (ALT and AST) levels, dramatic elevation in serum lactic dehydrogenase (LDH), and decrease in renal function. The WBC count was 19.4 × 10^9^/L; neutrophil percentage, 88.5%; hemoglobin concentration, 172 g/L; platelet count, 320 × 10^9^/L; BUN, 8.91–23.63 mmol/L; serum creatinine, 210–216 μmol/L; albumin (ALB), 42.4 g/L; LDH, 3159 U/L; creatine kinase (CK) 1459 U/L; ALT, 113 U/L; and AST, 113 U/L. Urinalysis revealed the urine protein score to be 3+; RBC, 3+; and WBC, 1+. Abdominal ultrasonography revealed a small right kidney and left kidney stone shadow without hydronephrosis. The patient underwent treatment with cephalosporins and amlodipine besylate (orally administered at 5 mg/d) without nonsteroidal anti-inflammatory drugs. Seven days ago, his follow-up examination indicated that his renal function did not show any significant improvement, with the serum creatinine level being 194 μmol/L and urine protein 2+, urine RBC 1+. With regard to his medical history, he had syncope a year ago, and his systolic blood pressure at the time was 180 mm Hg. However, he did not continue with the antihypertensive drugs or undergo further medical examination.

**FIGURE 1:**
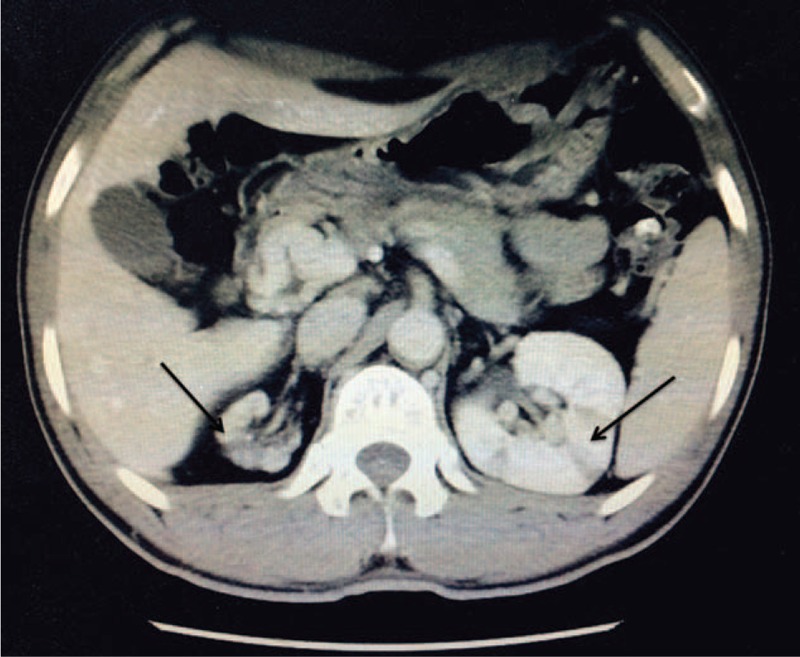
Computed tomography scan showing multiple areas with low-signal attenuation in the right and left renal parenchyma with right kidney shrinkage.

After the episode of acute pain in the left loin described above, this patient was admitted to our hospital with mild pain in the left loin. He was apyrexial and his blood pressure was 128/82 mm Hg. The complete blood count (CBC) was normal. The ALT level was 39 IU/L; AST, 19 IU/L; ALB, 41.6 g/L; BUN, 8.03 mmol/L; Scr, 151.20 μmol/L (normal range, 44–133 μmol/L); CK, 56 IU/L; LDH, 199 IU/L; and LDL-c, 3.19 mmol/L. Urinalysis showed that the protein score was 1+; RBC, 0.5/uL; and WBC, 51.6/uL. The 24-hour urine protein level was 0.74 g, and the urine bacterial culture was negative. He was negative for antinuclear antibodies, anti-dsDNA antibodies, anticardiolipin antibody, anti-beta 2-resistant GP1 antibodies, antineutrophil cytoplasmic antibodies, and antiglomerular basement membrane antibodies. The immunoglobulin (IgG, IgA, IgM), complement (C3, C4), and C-reactive protein (CRP) levels, and the erythrocyte sedimentation rate were normal. The fibrinogen and d-dimer levels were both unremarkable, and thrombelastography did not reveal any abnormalities. However, he was positive for lupus anticoagulant (dilute Russell viper venom time, dRVVT 1.28; [0.8–1.2] silica clotting time, SCT 1.37 [0.84–1.16]), and showed decreased protein C activity (52.10%; normal, 76%–158%), increased factor V coagulant activity (FVC) (145.10%; normal, 70%–130%), and a high homocysteine (HCY) level (67.32 μmol/L; normal, 5–17 μmol/L). Ultrasound images of the right renal artery trunk appeared obscure. A contrast-enhanced CT scan revealed the presence of multiple infarcts and calculi in the left kidney, segmental thickening in the abdominal aorta wall, and stenosis in the superior mesenteric vein trunk with chronic thrombosis and multiple collateral vessels (Figure 1). An ultrasonic cardiogram excluded left ventricular and aortic thrombi. Bilateral neck vascular ultrasonography indicated that the thickness of the intima media of the common carotid artery had increased. MRI indicated abnormal signal intensity (high intensity on T1 and T2 images and low intensity on T2FLAIR and DWI images) in the left and right cerebellum hemisphere, which suggested the presence of old ischemic lesions.

**FIGURE 2:**
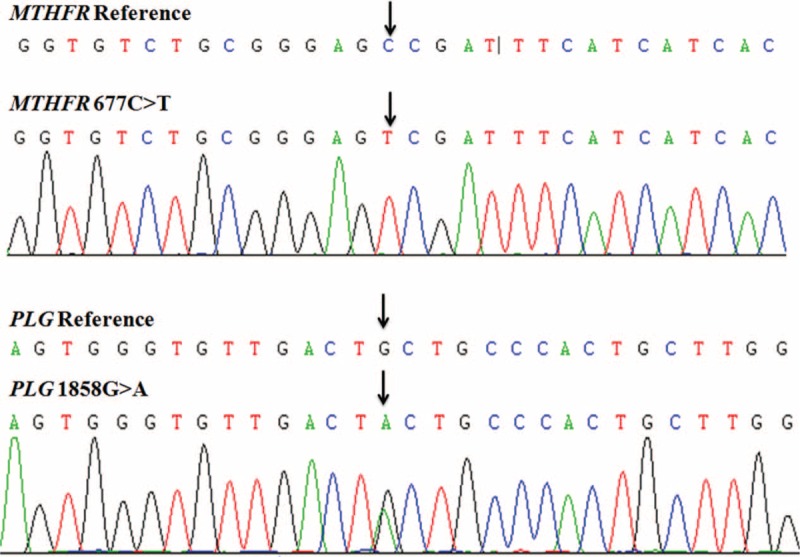
Identified mutations by next-generation sequencing were further verified by Sanger sequencing.

Analysis of genetic mutations was performed to check whether the patient had hereditary thrombophilia. Exome sequencing using the next-generation sequencing methods showed that the patient was homozygous for the *MTHFR* 677C>T mutation (*MTHFR* 677C>T occurs in exon 4 at the folate-binding site and results in the substitution of alanine with valine [A222V]) and heterozygous for the *PLG* 1858A>T mutation (*PLG* 1858G>A results in the substitution of alanine with threonine [A620T]). The mutations were further verified by Sanger sequencing (Figure 2).

The patient was commenced on life-long warfarin therapy, and INR was maintained between 2 and 3. Folic acid was recommended for reduction of the serum HCY level. At the three-month follow-up examination, his creatinine level was found to have returned to 130 μmol/L, which corresponded to an estimated GFR of 56 mL/min/1.73 m^2^. It indicated there was acute-on-chronic renal failure, rather than irreversible progression of CKD.

This study was approved by the Human Ethics Committee of Peking University First Hospital. Written informed consent was obtained from the patient for publication of the case report and any accompanying images.

## DISCUSSION

Here, we have reported a rare case of asynchronous bilateral RI and thrombophilia. The patient had been admitted to the hospital three times in the last two years with abdominal/flank pain, nausea, vomiting, and impaired kidney function. These entailed extra cost to the patient and also affected his quality of life; this case therefore highlights the importance of prompt diagnosis of RI.

It has been reported that the triad of pain, elevated LDH, and proteinuria and/or hematuria is observed in over 80% of RI cases.^4^ For differential diagnosis, renal pain or renal colic symptoms may be present in conditions such as renal colic (flank pain and hematuria), or acute pyelonephritis (flank pain and fever)—but neither nephrolithiasis nor pyelonephritis is associated with an elevation in serum LDH. Additional conditions that mimic the clinical manifestation of acute RI including mesenteric ischemia, and other causes of abdominal pain (i.e., cholecystitis and pancreatitis), may have different pain location, chronology, severity, aggravating/alleviating factors, and associated symptoms. A previous history of atrial fibrillation (or heart palpitation) or recent intra-arterial manipulation increases the likelihood of RI or mesenteric ischemia. Thus, it is in fact easy to differentiate RI from acute abdominal diseases. In addition to these indicators, contrast-enhanced renal CT can be used for rapid diagnosis:^7^ the classic finding is wedge-shaped perfusion defects. It has been reported that bilateral renal involvement is more frequent in patients with coagulation dysfunction, and that there is a considerable increase in the LDH concentration is patients with hypercoagulability; these findings are consistent with the current case.^8,9^ The hypercoagulability state, typically associated with vein thrombosis, is increasingly being associated with arterial vascular events.^4^ The patient in the present case had venous thrombus (superior mesenteric vein trunk stenosis with chronic thrombosis on contrast-enhanced CT scans) and multiple arterial embolic sites (right renal artery trunk, and segmental thickenings in the abdominal aorta wall and possibly skull and neck vessels) without evidence of left ventricular thrombus (by echocardiography) or a history of atrial fibrillation. The causes of unilateral small size kidney may be multiple, including previous renal infarction and atherosclerosis. But, renal manifestations may be different for these two circumstances. In atheroemboli, there are typically nondistensible, irregularly shaped, and smaller in size, tend to produce incomplete arterial occlusion of more distal vessels, with secondary ischemic atrophy rather than renal infarction. And common underlying diseases for renal artery injury including renal artery dissection, trauma, Marfan syndrome, and polyarteritis nodosa could be easily excluded.

Both inherited and acquired causes of hypercoagulability have been documented. The patient in the current report had several risk factors for thrombophilia, such as protein C deficiency, increased factor V coagulant activity (activated protein C resistance), hyperhomocysteinemia, presence of lupus anticoagulant, and smoking. The most frequent causes of an inherited (primary) hypercoagulability state are factor V Leiden mutations and prothrombin gene mutations, which together account for 50% to 60% of the cases.^2–6^ Defects in protein S, protein C, and antithrombin (formerly known as antithrombin III) account for most of the remaining cases, while dysfibrinogenemia is a rare cause.^10–12^ In the present case, no protein C or factor V mutations were found, based on which hereditary thrombophilia was excluded. However, the patient was homozygous for the *MTHFR* 677 C>T mutation, which is believed to be the strongest genetic determinant of plasma HCY concentration.^10^ Methylenetetrahydrofolate reductase (*MTHFR*) is the key enzyme for HCY metabolism: it catalyzes the conversion of 5,10-methyltetrahydrofolate to 5-methyltetrahydrofolate, a methyl donor in the remethylation of HCY. More importantly, it has been reported that homozygosity for *MTHFR* 677TT is more frequently associated with higher plasma HCY levels than heterozygosity for or absence of this mutation.^10^ In addition, the *MTHFR* 677 C>T mutation has been described as an inherited prothrombotic abnormality in some reports of renal venous thrombosis and RI. In renal failure patients, this polymorphism has a major effect on the HCY plasma concentrations.^10^ In addition, this patient was also heterozygous for the *PLG* 1858G>A mutation. It has been suggested that heterozygous mutations of the *PLG* gene result in dysfunctional plasminogen with decreased activity (“dysplasminogenemia”), which might be a predisposing factor for thrombotic events^11^ and atypical hemolytic uremic syndrome.^12^ Even though patients with dysplasminogenemia have a normal immunoreactive plasminogen level, the functional activity of plasminogen is significantly decreased due to abnormalities in the mutant PLG protein. Thus, gene mutation screening might help to further elucidate the underlying pathophysiology of RI, and future investigation of other identified or not-yet-identified genetic variants may be of interest. As this was a single case with multiple coagulation defects, with both arterial and venous thrombotic events (which is rare), these findings do not provide enough evidence for the mechanisms of RI and hereditary thrombophilia. Rather, this case demonstrates the complexity of thrombophilia, with both inherited and acquired risk factors interacting to produce a thrombotic phenotype.

**FIGURE 3:**
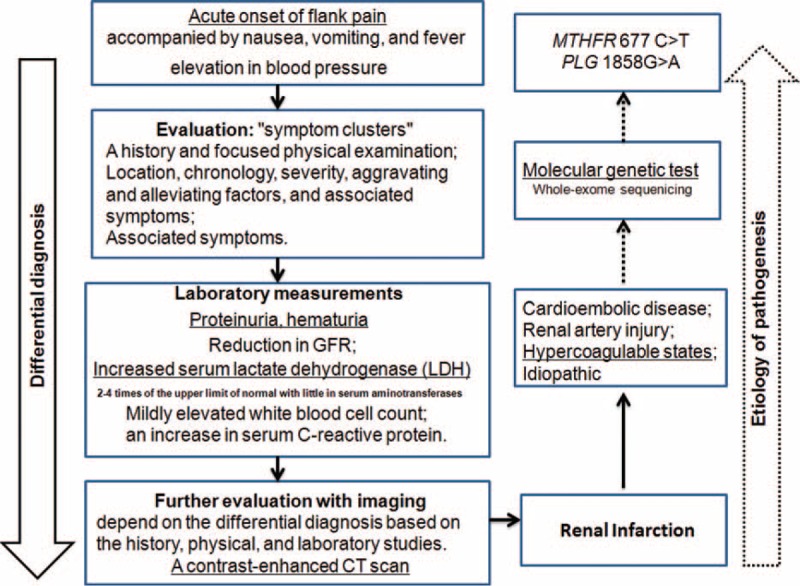
Current “walk through” pointing to the final diagnosis and etiology.

In summary, we describe a patient with bilateral RI with multiple risk factors for coagulation dysfunction (Figure 3, our “walk through” pointing to the final diagnosis). Genetic screening identified a possible causative mutation for hyperhomocysteinemia. Future more widespread study with more patients will be of highly merits.

## Supplementary Material

Supplemental Digital Content

## References

[1] Carey HB Boltax R Dickey KW Bilateral renal infarction secondary to paradoxical embolism. *Am J Kidney Dis* 1999; 34:752–755. 10516359 10.1016/S0272-6386(99)70403-8

[2] Kirchgatterer A Lugmayr H Aspock G Renal infarction due to combination of fibromuscular dysplasia and factor V Leiden mutation. *Nephrol Dial Transplant* 2004; 19:512–513. 14736990 10.1093/ndt/gfg536

[3] Chu PL Wei YF Huang JW Clinical characteristics of patients with segmental renal infarction. *Nephrology (Carlton)* 2006; 11:336–340. 16889574 10.1111/j.1440-1797.2006.00586.x

[4] Bourgault M Grimbert P Verret C Acute renal infarction: a case series. *Clin J Am Soc Nephrol* 2013; 8:392–398. 23204242 10.2215/CJN.05570612 PMC3586969

[5] Huang CC Lo HC Huang HH ED presentations of acute renal infarction. *Am J Emerg Med* 2007; 25:164–169. 17276805 10.1016/j.ajem.2006.06.010

[6] Wiles KS Hastings L Muthuppalaniappan VM Bilateral renal artery thrombosis in inherited thrombophilia: a rare cause of acute kidney injury. *Int J Nephrol Renovasc Dis* 2014; 7:35–38. 24465133 10.2147/IJNRD.S50948 PMC3900314

[7] Chung SD Yu HJ Huang KH Bilateral renal infarction. *Urology* 2009; 73:273–274. 18829084 10.1016/j.urology.2008.07.024

[8] Godfrey RL Clark J Field B Bilateral adrenal haemorrhagic infarction in a patient with antiphospholipid syndrome. *BMJ Case Rep* 2014; pii: bcr2014207050. doi: 10.1136/bcr-2014-207050. 10.1136/bcr-2014-207050 PMC4244402 25410037

[9] Mihout F Joseph L Brocheriou I Bilateral kidney infarction due to primary Al amyloidosis: a first case report. *Medicine (Baltimore)* 2015; 94:e777. 25929920 10.1097/MD.0000000000000777 PMC4603041

[10] Sunder-Plassmann G Fodinger M Genetic determinants of the homocysteine level. *Kidney Int Suppl* 2003; S141–S144. 12694331 10.1046/j.1523-1755.63.s84.52.x

[11] Schuster V Hugle B Tefs K Plasminogen deficiency. *J Thromb Haemost* 2007; 5:2315–2322. 17900274 10.1111/j.1538-7836.2007.02776.x

[12] Bu F Maga T Meyer NC Comprehensive genetic analysis of complement and coagulation genes in atypical hemolytic uremic syndrome. *J Am Soc Nephrol* 2014; 25:55–64. 24029428 10.1681/ASN.2013050453 PMC3871781

