# Supplementary material for: Asynchronous Bilateral Renal Infarction and Thrombophilia With Associated Gene Mutations in a 43-Year-Old Man: A Case Report
Source: Medicine (Baltimore). 2016 Apr 8;95(14):e3258. doi: 10.1097/MD.0000000000003258 (PMC4998791; doi:10.1097/MD.0000000000003258)
Supplement: Supplemental Digital Content [file medi-95-e3258-s001.doc]

**CASE REPORT**

**Gene mutations in a 43-year-old man with asynchronous bilateral renal infarction and thrombophilia by whole-exome sequencing analysis: A case report**

***Xu-jie Zhou†, MD & PhD , Li-jun Liu†, MD, Min Chen, MD & PhD, Fu-de Zhou, MD***

**Supplementary Table 1 Reported mutations for renal infarction.**

| Publication year | Origin of case | Gene Mutations | PubMed ID |
| --- | --- | --- | --- |
| 1996 | France | Factor V Leiden mutation | 8918725 |
| 2002 | France | MTHFR C677T | 11863127 |
| 2004 | Austria | Factor V Leiden mutation | 14736990 |
| 2004 | USA | MTHFR A1298C/C677T | 15696837 |
| 2008 | Spain | Prothrombin (G20210A) and MTHFR C677T | 18662160 |
| 2014 | UK | Pprothrombin (G20210A) | 24465133 |
| 2015 | USA | MTHFR C677T mutation | 25828971 |
| 2015 | Italy | Prothrombin and MTHFR | 26005936 |
